# The hoverfly and the wasp: A critique of the hallmarks of aging as a paradigm

**DOI:** 10.1016/j.arr.2021.101407

**Published:** 2021-07-13

**Authors:** David Gems, João Pedro de Magalhães

**Affiliations:** 1 Institute of Healthy Ageing, and Research Department of Genetics, Evolution and Environment, University College London, London WC1E 6BT, United Kingdom; 2 Integrative Genomics of Ageing Group, Institute of Ageing and Chronic Disease, University of Liverpool, Liverpool L7 8TX, United Kingdom

**Keywords:** aging, geroscience, hallmarks, paradigm, senescence, theory

## Abstract

With the goal of representing common denominators of aging in different organisms López-Otín et al. in 2013 described nine hallmarks of aging. Since then, this representation has become a major reference point for the biogerontology field. The template for the hallmarks of aging account originated from landmark papers by Hanahan and Weinberg (2000, 2011) defining first six and later ten hallmarks of cancer. Here we assess the strengths and weaknesses of the hallmarks of aging account. As a checklist of diverse major foci of current aging research, it has provided a useful shared overview for biogerontology during a time of transition in the field. It also seems useful in applied biogerontology, to identify interventions (e.g. drugs) that impact multiple symptomatic features of aging. However, while the hallmarks of cancer provide a paradigmatic account of the causes of cancer with profound explanatory power, the hallmarks of aging do not. A worry is that as a non-paradigm the hallmarks of aging have obscured the urgent need to define a genuine paradigm, one that can provide a useful basis for understanding the mechanistic causes of the diverse aging pathologies. We argue that biogerontology must look and move beyond the hallmarks to understand the process of aging.

*In its normal state, then, a scientific community is an immensely efficient instrument for solving the problems or puzzles that its paradigms define.* (Kuhn 1962) p. 166.

## Introduction

As the main cause of disease and death in the modern world, senescence (i.e. aging; not to be confused with replicative or cellular senescence; see glossary) is one of the major biological and medical challenges of the 21st century. It would therefore be invaluable to understand the central biological mechanisms of senescence and how they give rise to late-life disease, including cardiovascular disease, many forms of cancer, chronic obstructive pulmonary disease (COPD), dementia, and many other maladies. As the historian of science Thomas Kuhn describes in his classic book *The Structure of Scientific Revolutions*, mature fields of science possess effective paradigms, foundations of understanding that not only explain diverse phenomena but also engender a shared perspective for communities of working scientists (Kuhn 1962). For example chemistry has knowledge of atomic structure, the nature of chemical bonds, and the periodic table, while genetics has Mendel’s laws and the central dogma describing mechanisms of gene expression. Such mature paradigms possess great explanatory power enabling sustained scientific progress within their fields, leading to understanding of diverse phenomena, and multiple practical applications. Unfortunately, biogerontology, the study of the biology of aging, has yet to develop an effective central paradigm of this sort.

**Figure 1:**
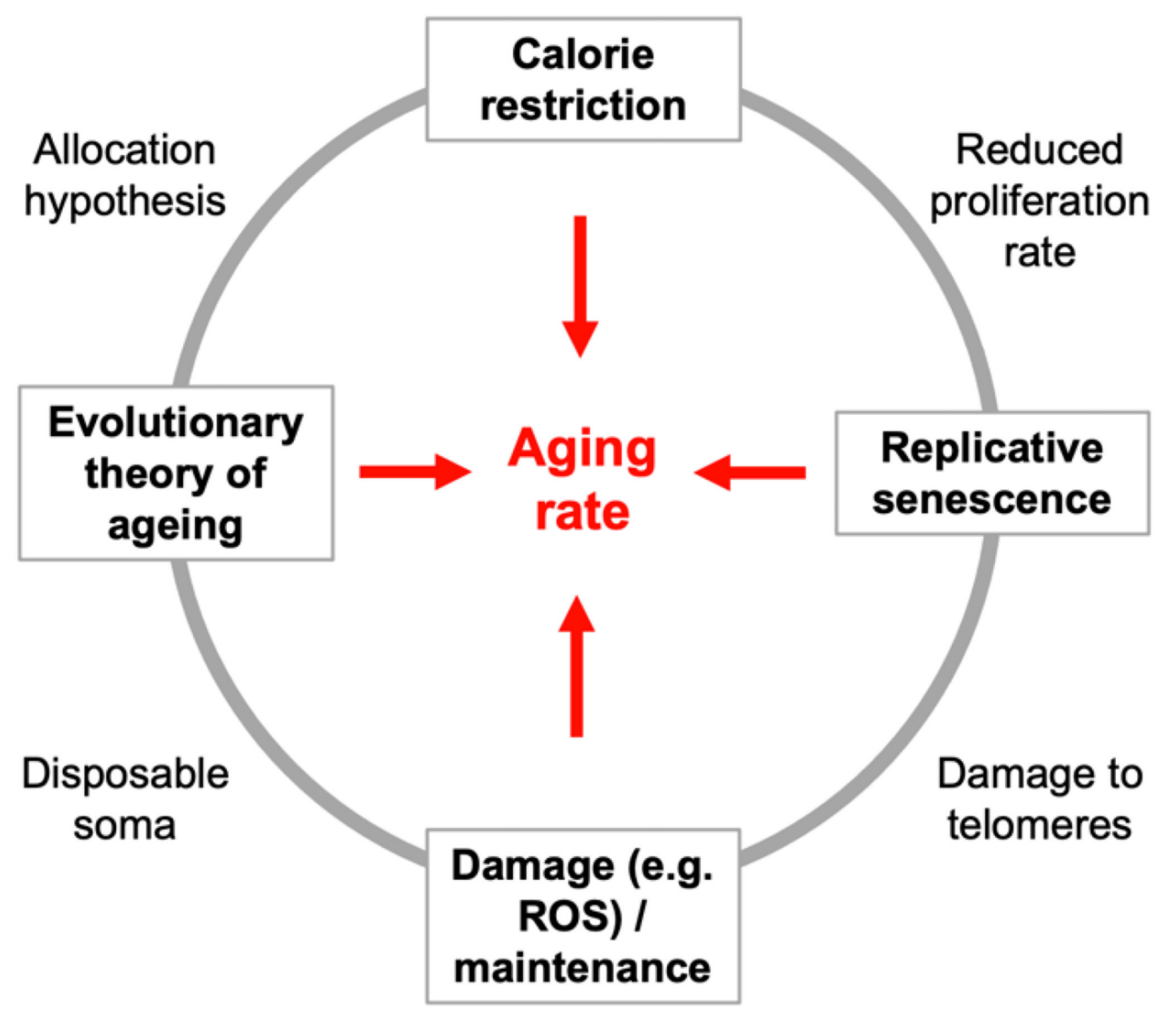
A very approximate representation of the traditional biogerontological conceptual framework. This framework, we argue, is experiencing a paradigmatic crisis. ROS, reactive oxygen species.

During the 1990s advances on many fronts led to the emergence of something resembling a broad paradigm for the biology of aging. This was formed from a set of observations and theories about aging, related to one another by further hypotheses that bound the framework together (Figure 1). Key elements were a belief that aging is a process of damage accumulation (Harman 1956; Kirkwood 2005; Hayflick 2007; Shore & Ruvkun 2013); that energy metabolism and mitochondrial function causes aging (Pearl 1928; Barja 2004); that the aging process as a whole can be slowed down in all organisms by caloric restriction (CR) (Masoro 1995); that replicative senescence caused by telomere shortening limits cell proliferation and lifespan (Hayflick & Moorhead 1961; Olovnikov 1996; Bodnar *et al*. 1998); and that the evolution of aging is due to the declining force of natural selection at later ages (Medawar 1952; Williams 1957; Hamilton 1966). Connecting ideas included the notion that production of reactive oxygen species (ROS) as a byproduct of mitochondrial respiration linked the metabolic and damage theories (Sohal & Weindruch 1996; Finkel & Holbrook 2000); that ROS causes DNA damage at telomeres leading to replicative senescence (von Zglinicki *et al*. 1995; von Zglinicki *et al*. 2001); and the disposable soma theory linked the evolutionary theory not only to the damage theory (Kirkwood & Holliday 1979; Kirkwood & Austad 2000), but also to CR through the energy allocation hypothesis, which argues that reduced energy availability leads to increased investment in somatic maintenance (Holliday 1989; Masoro & Austad 1996). Within this broad explanatory framework one component paradigm is particularly prominent: the idea that aging rate is a function of damage accumulation and somatic maintenance (the damage maintenance paradigm).

## An emerging paradigmatic crisis in the aging field

During the evolution of scientific fields there periodically occur scientific revolutions that are preceded by periods of crisis affecting their central paradigms and give rise to newer, more effective paradigms (Kuhn 1962). The aging field expanded significantly during the 2000s, leading to extensive and rigorous testing of prior ideas, and the emergence of new findings supporting alternative perspectives. This began a process of unravelling of the earlier framework that has been continuing ever since. For example, the notion of the importance of mitochondrial ROS as a cause of aging was a glue that held together a number of elements of the framework (Beckman & Ames 1998). But by the later 2000s accumulating evidence against this idea lead to serious doubts about it (de Magalhaes & Church 2006; Gems & Doonan 2009; Perez *et al*. 2009), raising questions about the centrality of damage accumulation in aging. This in turn raised doubts about the validity of the disposable soma theory (Blagosklonny 2010; Maklakov & Chapman 2019). Meanwhile, emerging drivers of aging, such as the wild-type mammalian (or mechanistic) target of rapamycin (mTOR) and insulin/IGF-1 signaling pathways, are more obviously involved with growth and development than somatic maintenance (de Magalhaes & Church 2005; Blagosklonny 2008; Gems & Partridge 2013). Evidence challenged the importance of ROS in telomere shortening and replicative senescence (Jagannathan *et al*. 2016), pointing instead to the importance in aging of in vivo accumulation of hyper-secretory senescent cells where neither a role of replicative senescence or telomere shortening is clearly implicated (Campisi 2013). Continuing advances in the genetics of lifespan and discovery of longevity pathways and drugs (de Magalhaes 2021) have not been matched by advances in understanding the causes and mechanisms of aging. Really, during the last two decades, biogerontology’s conceptual world view has taken a beating.

## Arrival of the hallmarks of aging

**Figure 2:**
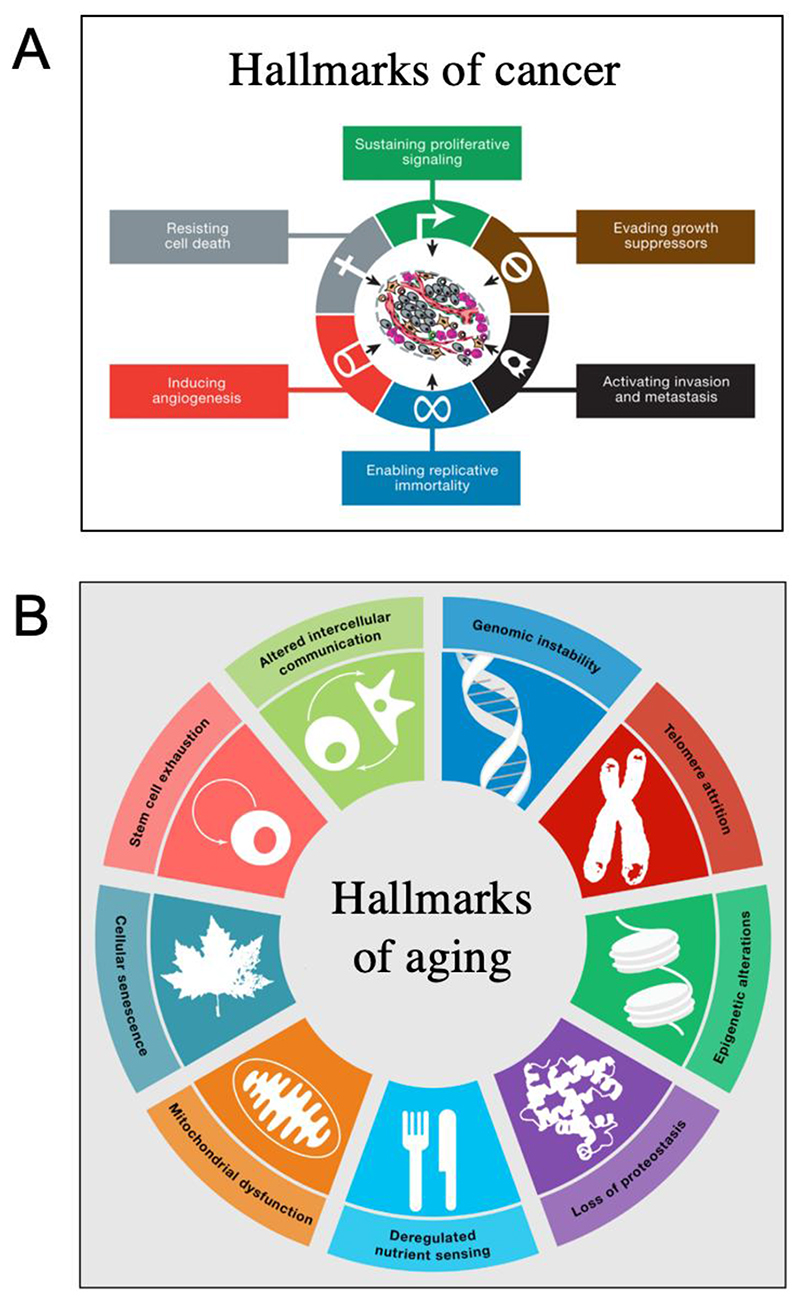
Hallmarks diagrams. A, Hallmarks of cancer (Hanahan & Weinberg 2011). B, Hallmarks of aging (López-Otín *et al*. 2013).

Since its publication in 2013, the hallmarks of aging is the most cited paper in the field of aging, amassing over 1,000 citations per year in recent years (Source: Google Scholar, accessed 12/05/2021). Inspired by an earlier account of hallmarks of cancer (Hanahan & Weinberg 2000; Hanahan & Weinberg 2011) (Figure 2A), the hallmarks of aging depict aging as resulting from nine causes: genomic instability, telomere attrition, epigenetic alterations, loss of proteostasis, deregulated nutrient sensing, mitochondrial dysfunction, cellular senescence, stem cell exhaustion, and altered intercellular communication. With increasing frequency in recent years, speakers will cite the hallmarks at the opening of their talk; for example, in a talk on molecular chaperones, the speaker will show the iconic hallmarks of aging figure (Figure 2B) and remind the audience that loss of proteostasis is one of the hallmarks of aging. In this way, they legitimise their research in terms of its relevance to aging, and locate themselves within a map of the biogerontological field, in which other biogerontologists in the audience can also locate themselves. Thus, like the central paradigm for a scientific discipline, the hallmarks representation serves the useful function of creating a shared perspective that unites the research community.

In addition to its utility in providing a shared perspective, and as a means for scientists to legitimize themselves as biogerontologists, another reason for the influence of the hallmarks account is that it provides an excellent overview of biogerontology, with authoritative accounts of many of its parts. For this reason, when talking to journalists confused by the aging field, or introducing the topic to students, the hallmarks of aging essay is a good review to recommend.

Key to biogerontology as a field is the observation that aging rate as a whole is plastic and amenable to manipulation and, therefore, that it is possible somehow to understand aging as a whole as a process. Key manifestations of plasticity in aging are the effects of CR, and of reduced insulin/IGF-1 and mTOR signaling, and evolutionary plasticity, as demonstrated by the large differences in aging rate among animal species, even in some cases between closely related species (e.g. our own species and our sibling species, the common chimpanzee; maximum lifespans, approximately 110 years and 60 years, respectively). One potential way to detect efficacy in interventions aiming to slow aging, including anti-aging drugs (Ermogenous *et al*. 2020), is to assay effects on multiple hallmarks of aging. A number of companies (e.g., Life Biosciences) have been set up to focus specifically on the hallmarks of aging. Thus, the hallmarks of aging provides an account that is both intelligible and attractive to the anti-aging industry, including investors, which has grown substantially in recent years (de Magalhães *et al*. 2017; de Magalhaes 2021). The list of hallmarks also provides a useful checklist for researchers studying specific diseases of aging, trying to understand their etiologies, and has generated further hallmarks lists, e.g. of the aging lung (Meiners *et al*. 2015), the aging brain (Mattson & Arumugam 2018), cellular senescence (Hernandez-Segura *et al*. 2018) and gene expression (Frenk & Houseley 2018).

The hallmarks of aging account is useful as a unifying perspective, but how useful is it as an explanatory paradigm? We informally canvased colleagues in the field for their views on this. All agreed that it provides a helpful overview of the field. However, many also expressed discontentment with it, in that it does not really provide an explanatory framework in terms of primary causes of aging. The hallmarks of aging idea seems to represent something more than just a list of things that are currently thought to be part of the broader aging process. The use of the hallmarks of cancer template, which describes a powerful explanatory paradigm, seems to imply that the hallmarks of aging does also. But is this really the case, i.e. does the hallmarks of aging account help scientists understand the underlying causes of aging in the way that a paradigm is supposed to? Is it a genuine paradigm at all?

## Measuring the hallmarks of aging against the hallmarks of cancer

In the original hallmarks of cancer essay, Hanahan and Weinberg express what they hope to achieve as follows: “One day, we imagine that cancer biology and treatment—at present, a patchwork quilt of cell biology, genetics, histopathology, biochemistry, immunology, and pharmacology—will become a science with a conceptual structure and logical coherence that rivals that of chemistry or physics” (Hanahan & Weinberg 2000). In fact, their account of cancer pathophysiology, incomplete though it may be, does provide not only a logically coherent conceptual structure, but one with a solid empirical basis. It is instructive to describe the hallmarks of cancer in a formal sense, i.e. in terms of its conceptual structure (rather than the specifics of what it actually says), and ask how far the hallmarks of aging corresponds to that structure.

**Figure 3:**
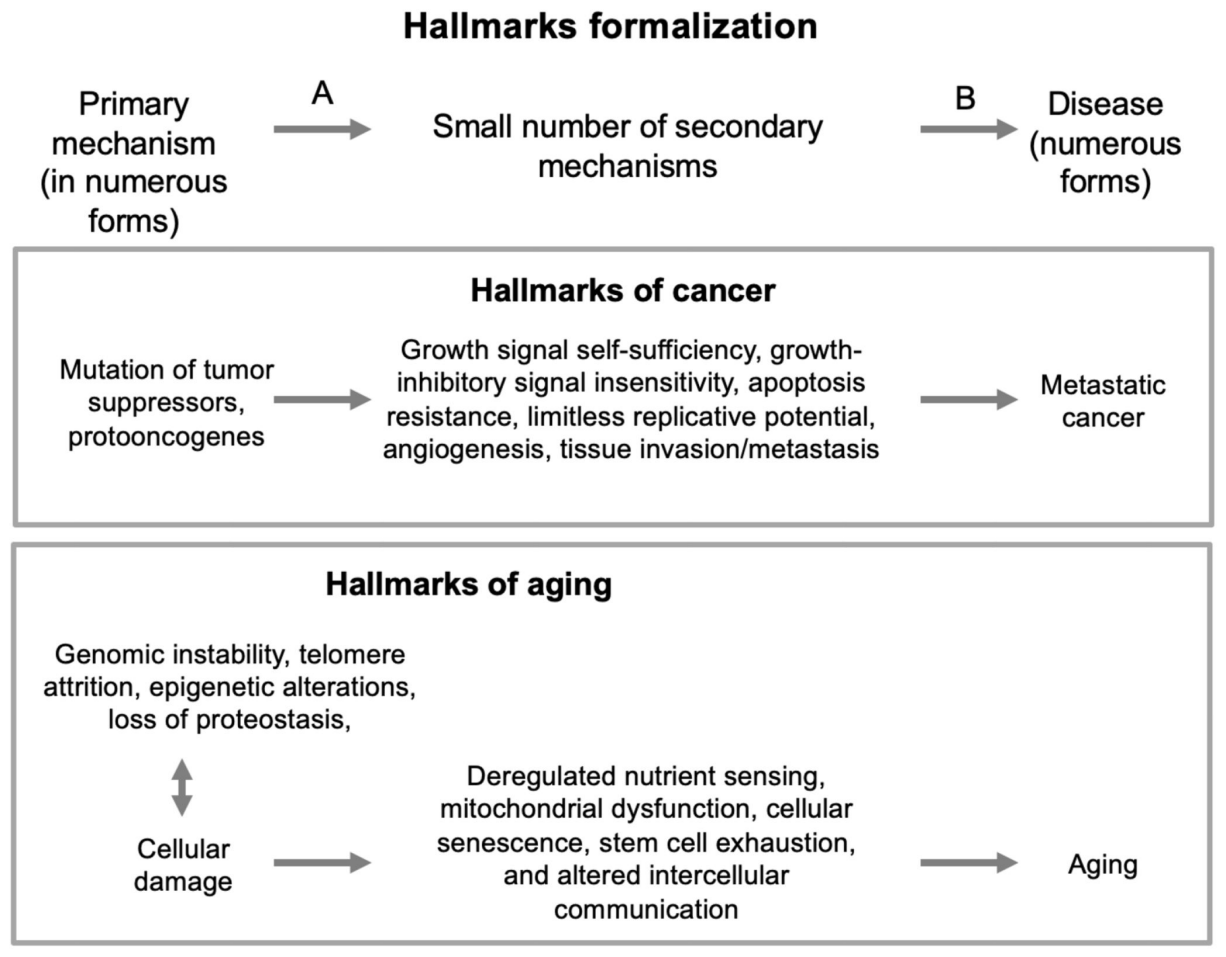
Formal representation of hallmarks of cancer conceptual structure (upper box). The hallmarks of aging approximate to this structure (lower box) (cf Figure 4).

Figure 3 sets out an approximate formalization of the hallmarks of cancer account. It is a description of the cause of cancer in two stages. According to this scheme, the first, primary cause of cancer (A) is mutation of tumor suppressor genes and proto-oncogenes. This gives rise to six types of alteration, all of which are required for the development of metastatic cancer. These alterations can be viewed as secondary causes (B) that arise from the primary cause. The primary cause (mutation) is not included in their hallmarks diagram (Figure 2B), perhaps because it is viewed as so self evident; it is there in the minds of all oncologists. What the hallmarks of cancer account achieves is to explain how a wide variety of different mutations give rise, in different ways, to a panoply of different forms of cancer via a small set of secondary changes. Importantly, the hallmarks of cancer provides a compelling and empirically supported account of both primary and secondary mechanisms. To a significant degree, it explains cancer and, importantly, it provides an explanatory paradigm of great utility for guiding successful research in oncology. As crucial elements in an explanatory framework the term “hallmark”, as used here, has a special meaning, implying a far greater significance than its common meaning, which is “a typical characteristic”.

**Figure 4:**
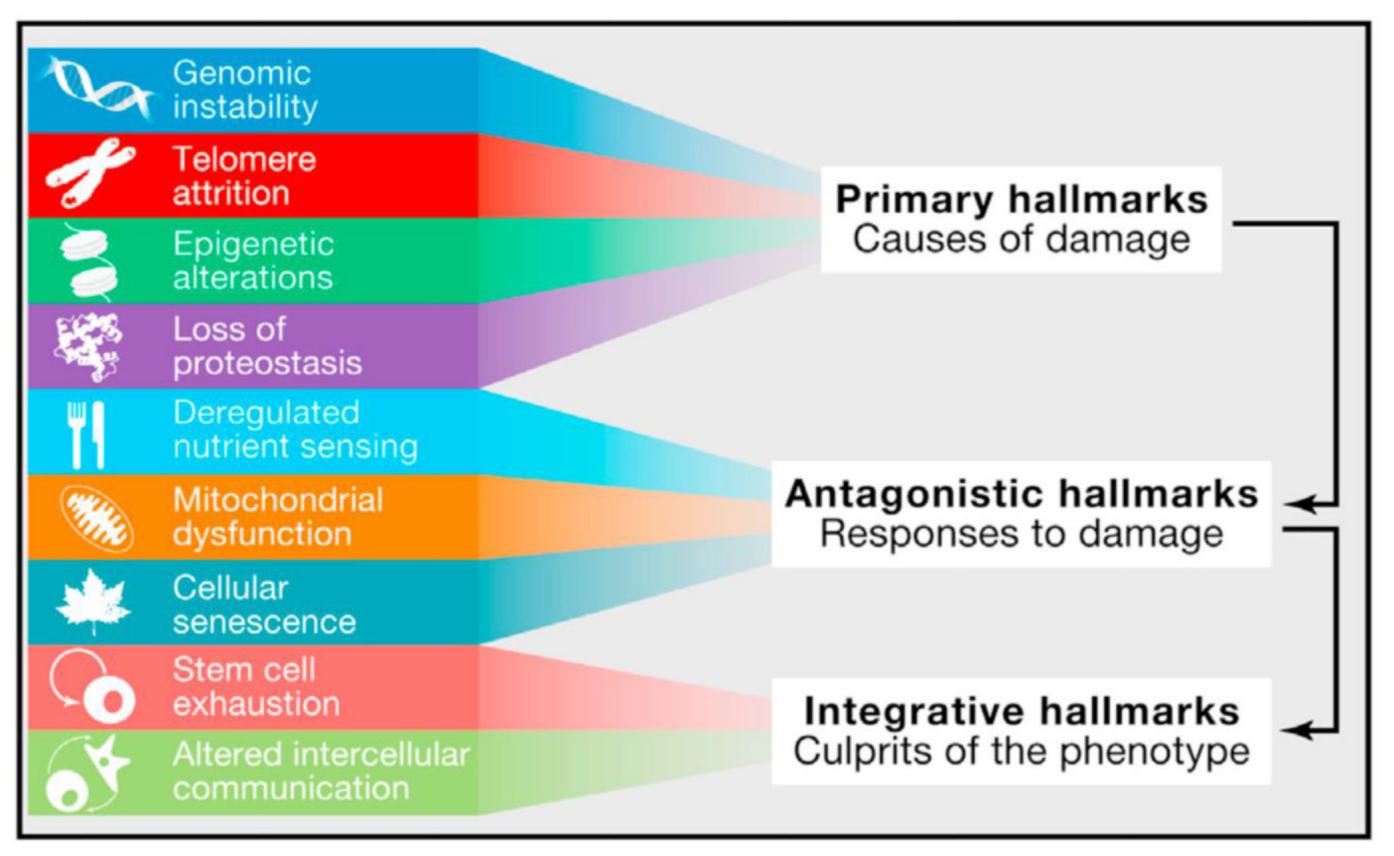
Conceptual structure from hallmarks of aging; Figure 6 from (López-Otín *et al*. 2013), in which it is specified that damage here refers to *cellular* damage.

How do the hallmarks of aging compare to the hallmarks of cancer in terms of an explanation of primary causes and secondary mechanisms? Although the hallmarks of aging review includes such an account, it is presented in only a brief and tentative way in the final Conclusions and Perspectives, the section of scientific articles where more speculation is traditionally permitted. The causal scheme presented (Figure 4) gives the impression of a quick improvisation or afterthought. Unlike the hallmarks of cancer, the hallmarks of aging include primary, secondary and tertiary mechanisms (Figure 3), classed as primary, antagonistic and integrative hallmarks. Several factors are represented as the causes of damage (specified as *cellular damage* in the figure legend), in a sense mirroring the multiple causes of somatic mutation in the hallmarks of cancer.

While the aging hallmarks scheme represents an original and brave attempt to restore order to a jumbled subject, if it is compared to the cancer hallmarks account a number of weaknesses stand out. (i) Cellular damage is assumed (as is traditional) to be the main causal common denominator of aging, but this is not certain. (ii) The list of hallmarks is somewhat arbitrary. (iii) Support for definition of hallmarks as primary vs secondary causes is sometimes lacking. (iv) Claims about how upstream causes give rise to downstream outcomes are often unproven. (v) How the secondary/tertiary causes give rise to aging is unclear. We will explore each of these issues in turn.

### i Cellular damage is assumed to be the causal common denominator of aging

In the hallmarks of cancer, whether or not mutation is the main, primary cause of aging is not discussed since it is a given. Following this template, the aging hallmarks account take it as given that cellular damage accumulation is the main driver of aging: “The time-dependent accumulation of cellular damage is widely considered the general cause of aging”. Yet at the time of writing, the crisis in the ROS theory (acknowledged in the hallmarks of aging essay) was raising doubts about this assumption (de Magalhaes 2005; Blagosklonny 2008; Gems & Doonan 2009; Perez *et al*. 2009; Lapointe & Hekimi 2010), and other primary causes of aging were already being discussed (de Magalhaes & Church 2005; Blagosklonny 2006; de Magalhães 2012; Gems & de la Guardia 2013; Gems & Partridge 2013).

However, the hallmarks of aging account more insinuates than states the centrality of damage as a primary cause; this may reflect concerns about the relative importance of molecular damage accumulation at the time. The use of the vaguer terms *damage* and *cellular damage* rather than *molecular damage* suggests a shift towards broader definitions of damage, c.f. Gladyshev’s concept of the deleteriome (Gladyshev 2016), perhaps reflecting uncertainty about the molecular damage theory.

We suggest that the influence of the hallmarks of aging account stems from its being a response to a field in transition (or crisis, as Thomas Kuhn would have it), particularly with respect to the utility of the damage maintenance paradigm for understanding aging’s causal mechanisms. It is true that the hallmarks of aging account departs from many earlier accounts of aging in not explicitly proposing stochastic molecular damage as the major, primary cause. Yet in a strange way its use of the hallmarks of cancer template (Figure 2A), in which the primary role of mutation is implicit, allows the threatened damage maintenance paradigm to retain a privileged and unchallenged (if rather ghostly) position within the conceptual scheme.

### ii The list of hallmarks is somewhat arbitrary

**Figure 5:**
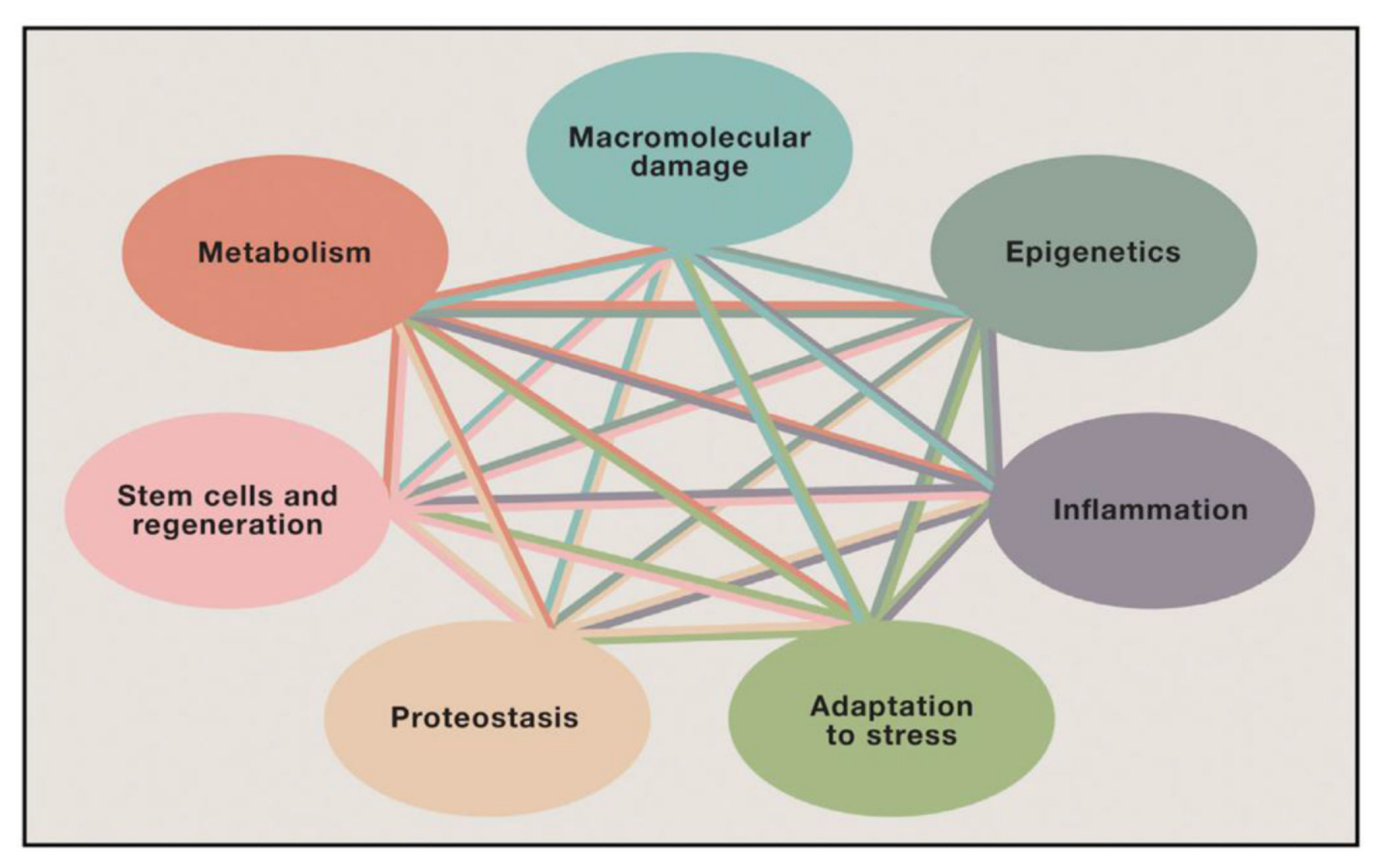
Seven pillars of aging (Kennedy *et al*. 2014). The identity of the pillars overlaps only slightly with the hallmarks. The interconnectedness depicted denotes a hopelessness with respect to an effective explanatory paradigm which is in sharp contrast to the hallmarks of cancer. Such interconnectedness is also argued to be present between the hallmarks of aging.

Considering the account of cancer etiology presented, the six hallmarks of cancer listed are each important for metastatic cancer development, and the six together go quite a long way to explain it. This is not the case for the hallmarks of aging. Biogerontology is in many ways still a relatively immature field, certainly far less mature than oncology. Like oncology prior to the emergence of the oncogene paradigm, it is characterised by a diversity of competing theories and approaches, which drift in and out of fashion. Other factors that could just as well have appeared on the list include antagonistic pleiotropy or trade-offs (Williams 1957; Partridge & Barton 1993), inflammation and inflammaging (Franceschi *et al*. 2000; Franceschi & Campisi 2014), consequences of mechanical senescence (Comfort 1964), altered endocrine function (Tatar *et al*. 2003; Kim & Choe 2019), programmatic changes and hyperfunction (de Magalhaes & Church 2005; Blagosklonny 2006; Maklakov & Chapman 2019), changes due to nutritional deficiency and excess (Kim & Choe 2019), immune senescence leading to increased infection (Aw *et al*. 2007; Fulop *et al*. 2013) and, if the list were being drawn up today, dysbiosis of the microbiome (Bana & Cabreiro 2019). Notably, of the 7 pillars of aging defined in another review published at around the same time (Kennedy *et al*. 2014) (Figure 5) only 3 of the hallmarks of aging are listed (proteostasis, epigenetics, stem cells and regeneration), though one can argue the two models overlap more as there are some similarities between other pillars and hallmarks. At earlier times, the hallmarks list might well have included oxygen free radicals as a byproduct of respiration (Beckman & Ames 1998), fast rate-of-living due to high metabolic rate (Sohal & Weindruch 1996), translation error catastrophes (Orgel 1963), and entropy (Hayflick 2007).

The factors listed as hallmarks are akin to placing names on a map of the biogerontology field, describing the interests of various groupings within the field, much as New York, Boston, Chicago and Los Angeles appear on the map of the USA. Inclusion on the map seems to reflect the more fashionable and better-funded areas. There also appears to be a bias away from those determinants that are more obviously of medical relevance (e.g. inflammation, immunosenescence, mechanical senescence); the neglect of age-related disease in the hallmarks of aging account is discussed below.

### iii Unsupported distinction of primary, secondary and tertiary causes

The causal chain in the hallmarks of aging account (Figure 4) is so tentative that it would be fitting to discount it as playful end-of-paper hand-waving, were it not for the fact that the article has been taken so seriously. In the cancer hallmarks account, DNA damage is a largely self-explanatory primary cause of cancer. This is not the case for some of the putative primary causes of aging. What causes epigenetic changes and loss of proteostasis? Are the mechanisms stochastic or programmatic? On the other hand, one could argue that *deregulated nutrient signaling* is a primary mechanism. The aging field was electrified by the discovery that single gene mutations can markedly extend lifespan in animal models, particularly the nematode *Caenorhabditis elegans* (Friedman & Johnson 1988; Brown-Borg *et al*. 1996; Kenyon 2010). Most notably, this led to identification of wild-type insulin/IGF-1/mTOR signaling as a cause of multiple diseases of aging and shorter lifespan in animal models (Garigan *et al*. 2002; Powers *et al*. 2006; Tsang *et al*. 2007; Selman *et al*. 2008; Kapahi *et al*. 2010). Here wild-type gene action and signaling is clearly identified as a primary cause of aging, not the deregulation of these pathways, and certainly not deregulation resulting from damage. In their representation of deregulated nutrient signaling as a secondary cause of aging driven by damage, the aging hallmarks account seems to shoe-horn evidence to fit the cancer hallmarks template, with its overarching primary cause (here damage).

### iv Claims about how secondary causes arise from primary causes are often unproven

In the hallmarks of cancer account, secondary causes involve cellular changes resulting from mutation. For example, mutation-induced hyper-activity of the *bcl-2* oncogene leads to apoptosis resistance, promoting survival and proliferation of cancer cells (Hanahan & Weinberg 2000). The account of emergence of a secondary cause from a primary one provides a relatively full and well-supported explanation. By contrast, that all the hallmarks of aging result as the direct or indirect consequences of cellular damage is doubtful. As a claim about individual hallmarks, this attribution ranges from highly plausible to highly implausible. For example, it is self-evident that DNA damage is a major cause of genome instability, and it is a major determinant of telomere shortening. It is also plausible that damage accumulation plays a role in epigenetic alterations, loss of proteostasis, mitochondrial dysfunction and cellular senescence.

However, in many of these cases, it is not clear that damage is the predominant cause leading to aging in vivo. For example, programmatic changes during adulthood are likely to drive epigenetic changes (de Magalhaes & Church 2005; de Magalhães 2012; Blagosklonny 2018; Horvath & Raj 2018) and in *C. elegans* at least, collapse of proteostasis (Labbadia & Morimoto 2015). While there is ample evidence that damage accumulation promotes replicative senescence in vitro (von Zglinicki *et al*. 1995; de Magalhaes & Passos 2018), its relative importance as a cause of senescent cell accumulation in vivo is unclear, and various other determinative factors are clearly operative, including paracrine senescence and reduced efficiency of clearance due to immunosenescence (van Deursen 2014; Burton & Stolzing 2018; de Magalhaes & Passos 2018).

### v How the secondary/tertiary causes give rise to aging is unclear

**Figure 6:**
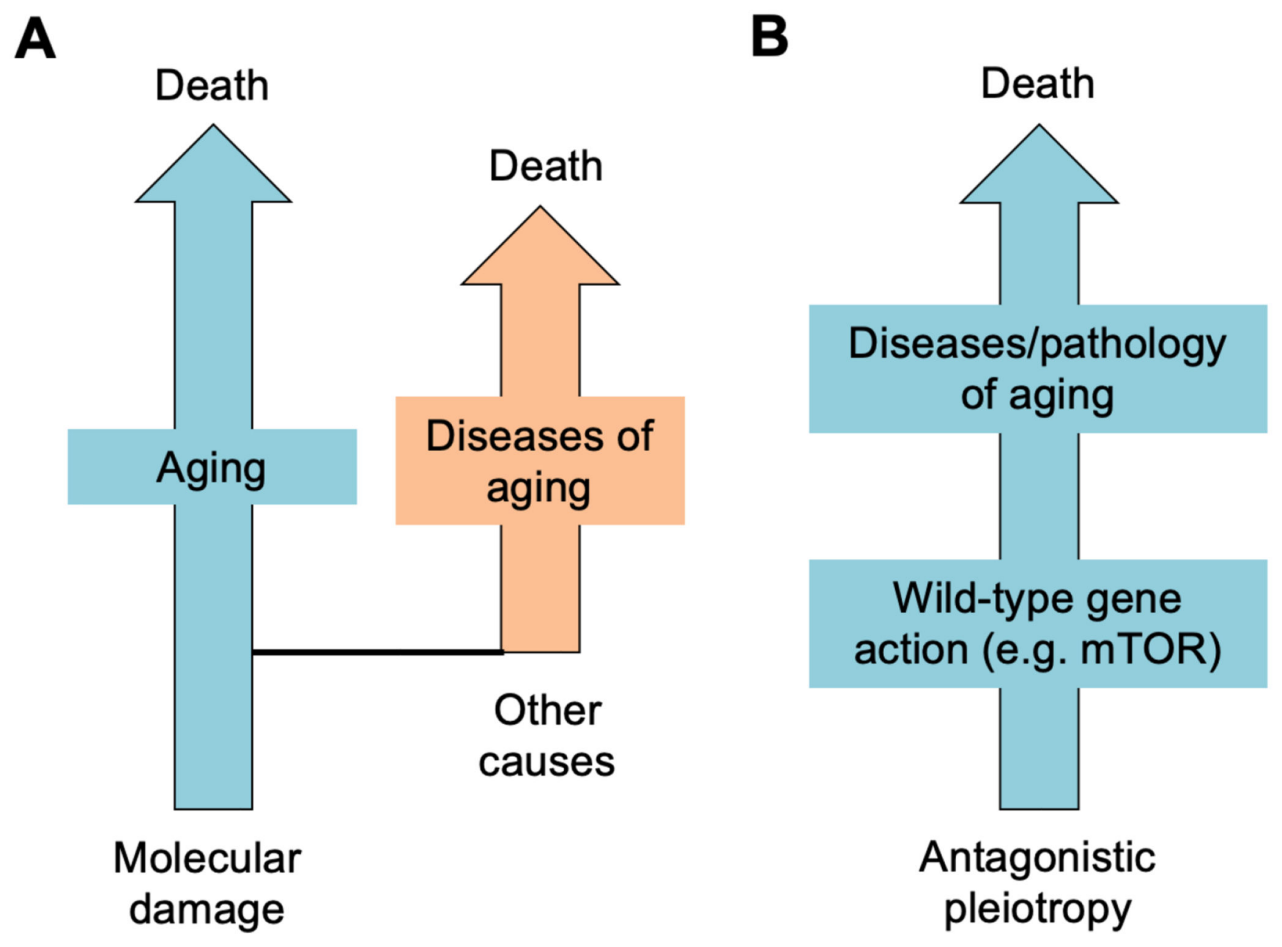
The false distinction between aging and disease; adapted from (Blagosklonny 2007). A, Aging as a process distinct from late-life disease; here disease is incidental to understanding aging. B, Aging as diseases/pathologies caused by wild-type gene action; here disease/pathology is critical to understanding aging.

The hallmarks of aging scheme fails most seriously where the hallmarks of cancer triumphs: in explaining how secondary causes combine to generate disease. Arguably, one reason for this is a lack of clarity about what aging means. An affliction of the biogerontology field is an odd view of the relationship between aging, late-life disease and death. Senescence manifests as declining health and death due to diverse pathologies. Some senescent changes are plainly identifiable as diseases (e.g. colon cancer, stroke, macular degeneration) while others involve subtler forms of degenerative change (e.g. sarcopenia, osteoporosis, skin senescence) whose combined effects can cause rapid demise among elderly people fortunate enough to escape the lethal diseases. Yet a traditional view in biogerontology is that aging is a process apart from senescent pathology, upstream of and determinative of it, and arising from deterioration at the subcellular level (e.g. damage from mitochondrial ROS, telomere shortening, protein aggregation). According to this view, two major factors cause mortality in later life, a major one, aging, and a minor one, aging-related diseases for which the underlying aging process is a risk factor (Figure 6). It has been argued that this misconception has misguided the field (Blagosklonny 2007; Gems 2015; Gladyshev & Gladyshev 2016), and led to a neglect of what should be the key question in biogerontology: how can etiologies of senescent pathology be understood in terms of underlying mechanisms of aging?

True to tradition, the hallmarks of aging account makes relatively little reference to specific pathologies of aging. Although some diseases are mentioned, including atherosclerosis, Alzheimer’s and Parkinson’s disease and cataract, many are not, such as COPD, osteoporosis, osteoarthritis, rheumatoid arthritis and stroke, and although cancer is discussed, specific cancers (e.g. breast, colon, prostate, lung, leukemia) are not. More importantly, there is little discussion of how hallmarks of aging give rise to diseases of aging or, critically, how multiple diseases of aging can be understood as originating from a small number of hallmarks. This is in sharp contrast to the hallmarks of cancer account.

In some ways, neglect of diseases of aging and the belief in damage as the main cause of aging are mutually supportive, since it is evident that factors other than damage are major determinants of many diseases of aging. To mention just a few examples, obesity contributes to cardiovascular disease (hypertension, atherosclerosis), cancer and type II diabetes (Aune *et al*. 2016); inflammatory hyperactivity to these, and to Alzheimer’s disease (Xia *et al*. 2016); and immunosenescence to increased infection, both new and recrudescent (e.g. herpes zoster [shingles]) (Fulop *et al*. 2016).

Also inconvenient to the scheme is the robust evidence that damage plays relatively little role in aging in *C. elegans* or its remarkable lifespan plasticity, and the importance of that to the premise that aging as a whole can be manipulated (Gems & Doonan 2009; Van Raamsdonk & Hekimi 2010). Overall, little reference is made to work on *C. elegans* (as a declaration of interest, both authors of this article work with *C. elegans*). In the context of model organisms, another limitation of the hallmarks of aging compared to the hallmarks of cancer is that the latter are strongly grounded in extensive human data. By contrast the former are based largely on extrapolations from studies in animal models, particularly short-lived models that may or may not be representative of human biology (de Magalhaes 2014).

While the role of all of the cancer hallmarks in cancer progression is compelling, this is not the case for some of the aging hallmarks and senescent pathology. For some the current evidence is quite good, and genomic instability due to defects in DNA repair can lead to segmental progeroid syndromes in mice and men (Freitas & de Magalhaes 2011; Niedernhofer *et al*. 2018; Schumacher *et al*. 2021). By contrast, epigenetic alterations are not known to be causal in mammalian aging or any specific senescent pathology, although they are strongly correlated with aging (Horvath 2013; Horvath & Raj 2018) and manipulating genes affecting chromatin status can increase lifespan in invertebrates (Benayoun *et al*. 2015). Moreover, while telomere attrition is listed as a hallmark of aging, human epidemiological data suggest a causal role of both short and very long telomeres in cancer, heart disease and other age-related diseases (Codd *et al*. 2013).

Another aspect of the definition of aging that is left vague is whether it can be regarded as a single process, as suggested by those interventions that appear to alter aging as a whole, and consistent with the idea that aging rate is a function of somatic maintenance processes that are genetically regulated. While this is implicit in the hallmarks of aging account, there is also an implicit worry that single cause explanations of aging cannot be valid (although cellular damage is presented as the main primary cause) (Figure 4).

An alternative view is that interventions that markedly extend lifespan do not do so by slowing the overall aging process, but rather by inhibiting determinants of multiple senescent pathologies - or etiologies of multimorbidity (Gems 2015). Potential examples here are mTOR hyper-activity specified by wild-type gene function (Blagosklonny 2006; Tsang *et al*. 2007), and senescent cell accumulation (Childs *et al*. 2017). The senescent multimorbidity perspective which, arguably, brings biogerontology closer to medical reality, is starting to be adopted (Ermogenous *et al*. 2020).

## The hoverfly and the wasp: pseudo-hallmarks and pseudo-paradigm

In Hanahan and Weinberg’s account, hallmarks as defined have considerable explanatory power: a small number of features whose combined action can make sense of great diversity in both upstream primary mechanisms (mutations affecting many genes) and downstream disease (cancer types and subtypes). As discussed here, the 9 topics within the aging field listed are not hallmarks in the Hanahan and Weinberg sense. By using the hallmarks of cancer template, which describes a true paradigm, the aging hallmarks account takes a somewhat arbitrary set of popular ideas from the aging field and, seemingly, dresses them up as a paradigm, even though a genuine paradigm as present in the hallmarks of cancer account does not exist in aging. This resembles an exercise in mimicry: as the hoverfly mimics the wasp to fool predators into believing that it has a sting, the hallmarks of aging puts on a resemblance to the hallmarks of cancer, to give the impression of a paradigm where one does not exist.

This is reflected not only in the (oblique) claim of one main primary cause leading to diverse consequences via a small number of essential determinants, but also the use of a similar title, a graphically similar hallmarks cartoon involving a ring with different colored sections each bearing a graphic representation (Figure 2A, B), published in the same journal (*Cell*). The widespread uptake of the hallmarks scheme suggests that the mimicry has been highly successful (though this was not, we believe, the deliberate intention of its authors). The 7 pillars of aging, a scheme from around the same time as the hallmarks of aging, similarly listed various aspects of aging but without the paradigmatic mimicry (Kennedy *et al*. 2014) (Figure 5); possibly in part due to this lack of paradigmatic allure the 7 pillars are rarely used to introduce talks and attract markedly fewer citations per year (~200).

The hallmarks of aging is now widely viewed as a central concept within biogerontology, even by some as a dogma that underpins the field. This is problematic for several reasons. First, the hallmarks of aging incorrectly gives an impression of an understanding of aging that does not exist; in fact it masks what is actually a crisis affecting traditional biogerontological paradigms relating to the mechanistic understanding of aging, particularly the primacy of damage. Second, a true paradigm provides a framework of understanding that guides research into understanding the subject that it addresses. In terms of understanding the causes of aging (in the same way that Hanahan and Weinberg seek to understand the causes of cancer) the hallmarks of aging scheme has relatively little to offer, except as a review of different aspects of the aging field (and as such, it is excellent). This is because it is not a paradigm in the proper sense. Rather, the hallmarks of aging, as a pseudo-paradigm, risks acting as a conceptual obstacle to the development of a genuine paradigm with real explanatory power.

## What might an operative paradigm look like?

Having thus far expended many critical words on the hallmarks of aging as a paradigm, can we say something constructive in terms of what a genuine paradigm might look like, at least in a very general way? Suggested shortcomings of the hallmarks of aging account include its implicit assumption that damage is the main primary cause of aging; its neglect of aging-related disease, arising from the aging-disease false dichotomy (Gems 2015); and the implicit assumption that there is such a thing as aging as a whole (i.e. interventions that extend lifespan slow the entire aging process).

The hallmarks of cancer template, as formalized (Figure 3) contains two particularly useful elements: the distinction between primary and secondary causes, and its account of how many forms of the primary cause (different mutations) can lead through a small number of types of change (the hallmarks) to highly diverse disease outcome (the many forms of cancer). But it is the second element that is so distinctive about the hallmarks template; the distinction between primary and secondary causes is a general feature of pathophysiology. As we discuss, the hallmarks of aging account at one point vaguely follows this template, defining damage as a predominant causal factor (Figure 4), though primary and second mechanisms are not well defined.

The problems with prior attempts to understand aging as the result of molecular damage, and the growing understanding of programmatic drivers of senescence, argue for a more multifactorial view of aging in terms of distinct types of *primary* mechanism. Taking senescence (as an outcome, rather than as a process) to be the sum of late-life pathologies, it is notable that most diseases of aging are multifactorial disorders, i.e. they result from multiple primary causes. This includes cardiovascular disease, Alzheimer’s disease, COPD, osteoporosis, osteoarthritis and many others (Glocker *et al*. 2006; Huertas & Palange 2011; Armstrong 2013; Glyn-Jones *et al*. 2015; Al Anouti *et al*. 2019; Higashi *et al*. 2019).

If the multiple causes of diseases of aging include multiple, distinct classes of primary cause, then the hallmarks of cancer template is not fitting to explain aging. In fact, there is already little doubt that senescence is the result of multiple, distinct primary causes. While understanding such primary causes remains a challenge for biogerontology and biomedical research more broadly, there are grounds for optimism that they may be so limited in number as to allow tractability in terms of mapping out senescent pathophysiology and designing treatment.

Likely primary determinants include the following. First, the main classes of primary cause of pathology in earlier life: mechanical damage (injury; c.f. mechanical senescence), molecular damage (including inherited and acquired mutations) and infectious pathogens. Second, the far less well understood classes of mechanism by which the wild-type genome determines aging rate. As a very broad approximation one may view the wild-type genome as a primary cause. However, it is clear that the wild-type genotype controls aging not only through all the standard etiological categories (affecting susceptibility/resistance to mechanical damage, acquired molecular damage, and infection) but also modes of action more specific to aging, such as the consequences of antagonistic pleiotropy (including trade-offs) (Williams 1957; Partridge & Barton 1993; Nesse & Williams 1994), programmatic mechanisms including futile program run-on (quasi-programs) (de Magalhaes & Church 2005; Blagosklonny 2006; Maklakov & Chapman 2019), costly programs (Gems *et al*. 2020), and consequences of biological constraint (Acerenza 2016).

**Figure 7:**
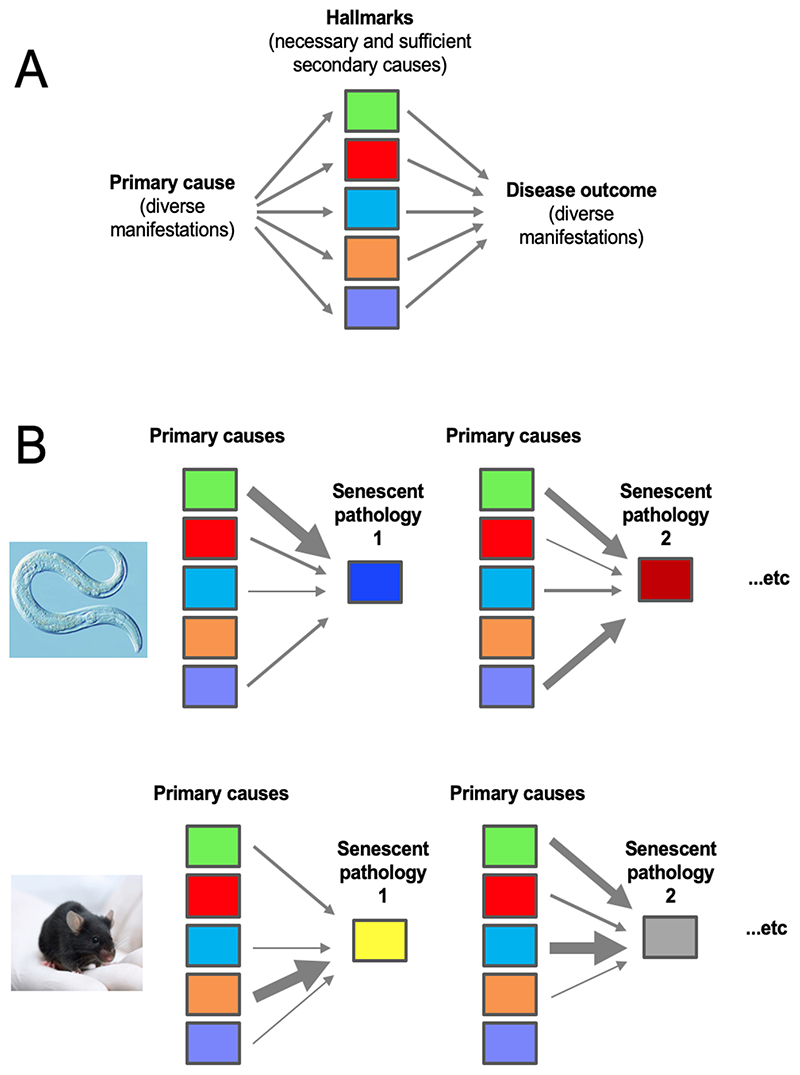
Alternative paradigm templates. A, the hallmarks template (single primary mechanism). B, multiple primary mechanisms template. Possible primary mechanisms include mechanical senescence, molecular damage, infectious pathogens, and wild-type gene action. According to this model, individual diseases of aging throughout the animal kingdom may be understood in terms of different combinations and extents of primary causes. The overall contribution to senescence varies between taxa. For example, the green box could represent programmatic aging mechanisms specified by wild-type gene action, and the orange box molecular damage (particularly DNA damage). According to this model, development of senescent pathology throughout the animal kingdom, however alien from human diseases, are governed by the same underlying set of principles of senescent pathophysiology. Arrow thickness represents relative pathophysiological contribution.

Thus, for aging a multi-cause template is needed rather than a single cause one (Figure 7). In this type of template, there are multiple classes of primary cause, which combine in different ways and contribute to differing extents to generate specific diseases of aging. We suggest that the basic set of broadly defined primary causes (or principles of senescent pathophysiology) is universal across the animal kingdom, and that their relative importance in aging differs between animal taxa and even between tissues in the same species. For example, DNA damage is an important driver of cancer in mammals (Niedernhofer *et al*. 2018), but appears to play little role in aging in *C. elegans* (Johnson & Hartman 1988). Since senescence is vastly more complex than cancer (which to a large extent is just one element of senescence), it is not possible to define secondary determinants in a useful way for aging as a whole, as is possible with hallmarks of cancer. However in a given animal taxon, for specific diseases of aging this is possible and useful - as shown by the hallmarks of cancer. This may extend also to senescent multimorbidity syndromes.

## Concluding remarks

The Hallmarks of Aging review has strengths and weaknesses. It provides an excellent overview of the biogerontology field, and both of us recommend it to students for this reason. While it has been useful for stimulating various anti-aging and longevity companies, it remains to be seen whether targeting the hallmarks is a sound approach for developing anti-aging therapies. However, the manner in which it uses the hallmarks of cancer as a template risks creating a false impression of a field with an explanatory paradigm. In fact, the traditional framework of mechanistic theories that has guided biogerontology for 30 years is in a state of paradigmatic crisis and transition. In this context, the scheme is akin to a folding screen bearing a hallmarks of aging diagram that blocks the view of the true state of undress of the field. Shivering behind the screen is the ailing damage maintenance paradigm. We argue that as a field we must look and move beyond the hallmarks to understand the process of aging. Only by doing so can paradigms be formulated that possess sufficient explanatory power to enable treatments for human aging to be developed.

## Glossary

Aging: Progressive changes in organisms during the course of adulthood, particularly deteriorative changes (see *senescence*), that arise largely from endogenous mechanisms.
Aging-disease false dichotomy: A fallacy, arguably, that views the process of aging as non-pathological, and distinct from age-related disease.
Allocation hypothesis: This proposes that caloric restriction increases lifespan by redirecting resource investment from reproduction into somatic maintenance (see disposable soma theory).
Antagonistic pleiotropy (AP): Where action of a given gene is both beneficial and detrimental to fitness. If the latter occurs later in life and is therefore subject to weaker selection, such a gene may be favored by natural selection, and promote aging.
Costly program: A biological program that simultaneously promotes fitness and incurs a cost in terms of pathological changes to tissues or organs where the program is executed. One form of programmatic mechanism involving hyperfunction by which AP causes senescence (cf. quasi-program).
Damage maintenance paradigm: The view that aging is caused by molecular damage accumulation, and longevity assured by mechanisms of somatic maintenance (e.g. the oxidative damage theory).
Deleteriome: The sum of all forms of damage and deterioration in an organism.
Disposable soma: Theory proposing that natural selection favors investment of limited resources into reproduction rather than somatic maintenance, accelerating damage accumulation and, therefore, senescence.
Etiology: The cause, set of causes, or manner of causation of a disease or condition.
Geroscience: The science of aging, and of aging-related disease.
Hyperfunction: Where wild-type gene function actively leads to senescent pathology, as opposed to passive random damage or wear and tear.
Longevity: The period of time an organism is expected to live under ideal circumstances..
Mechanical senescence: Senescence caused by activity-dependent wear and tear, e.g. abrasion of teeth, damage to cartilage in articular joints contributing to osteoarthritis.
Programmatic aging: Where complex biological processes contributes to senescence, but not necessarily to fitness (cf. quasi-programs, costly programs).
Run-on: Futile continuation of gene function or processes in later life, leading to pathology (cf. quasi-program).
Senescence: The overall process of deterioration with age or the resulting pathological condition (not to be confused with *cellular senescence,* which is a particular form of cell cycle arrest affecting some vertebrate cell types). Although aging has several meanings, in the biological context it is usually synonymous with senescence.
Senescent multimorbidity: Multiple forms of senescent pathology, which can arise from a common etiology, or etiologies.
Stochastic molecular damage: Molecular damage arising in a random, probabilistic fashion (e.g. DNA damage that accumulates in adult somatic tissues).
